# Cryptococcus infection in a non-HIV patient: a case report

**DOI:** 10.3402/jchimp.v2i3.19254

**Published:** 2012-10-15

**Authors:** Charmian D. Sittambalam, Heidi Hanna, Justin Martello, Dimitra Mitsani

**Affiliations:** Department of Internal Medicine, Union Memorial Hospital, University of Parkway, Baltimore, MD, USA

**Keywords:** cryptococcal infection, chronic steroid use, cryptococcal meningitis

## Abstract

Cryptococcal infections are fungal infections most commonly seen in immunocompromised patients. Chronic high-dose steroid may precipitate such an immunocompromised state and thus create susceptibility to fungal infections. *Cryptococcus neoformans* is a saprophyte usually found in soil contaminated with pigeon droppings. Suspicion to diagnose begins with clinical symptoms that can be non-specific such as fevers, cough, and headaches. We present a case of steroid-induced cryptococcal infection in a non-HIV-infected person.

*Cryptococcus neoformans* is an opportunistic infection seen in immunocompromised patients and is the fourth most common infection in AIDS patients with CD4 counts of less than 100. It is an encapsulated fungal organism that can affect the central nervous system (CNS) with pulmonary manifestations usually being among the first presenting signs.

Chronic steroid use can predispose patients to many infections. High steroid doses can decrease migration and inhibit chemotaxis of neutrophils, inhibit phagocytosis and intracellular killing, and decrease production of proinflammatory cytokines (1).

## Case report

A 63-year-old male with a medical history of diabetes mellitus, chronic obstructive pulmonary disease, hepatitis C, remote intravenous drug use, gastroesophageal reflux disease, and high-dose chronic steroid use for 5 years presented to the ER with severe headaches, a history of gait instability and weakness when standing, as well as a 40-pound weight loss over a 5- to 6-week period. He described the headaches as sharp, debilitating, and localized to the posterior skull and radiating to the top of his head. He described an inability to stand without collapsing prior to admission. He also reported contact with a large pigeon population in the area where he works.

Oral temperature was 37.4°C, heart rate 88, blood pressure 122/74, respiratory rate 20, and oxygen saturation of 96% on room air. The patient exhibited a cushingoid facies and marked central obesity, with signs of previous injection drug use as evidenced by multiple areas of scarred skin, now healed. He displayed completely normal neurologic functioning and was alert and oriented.

**Fig. 1:**
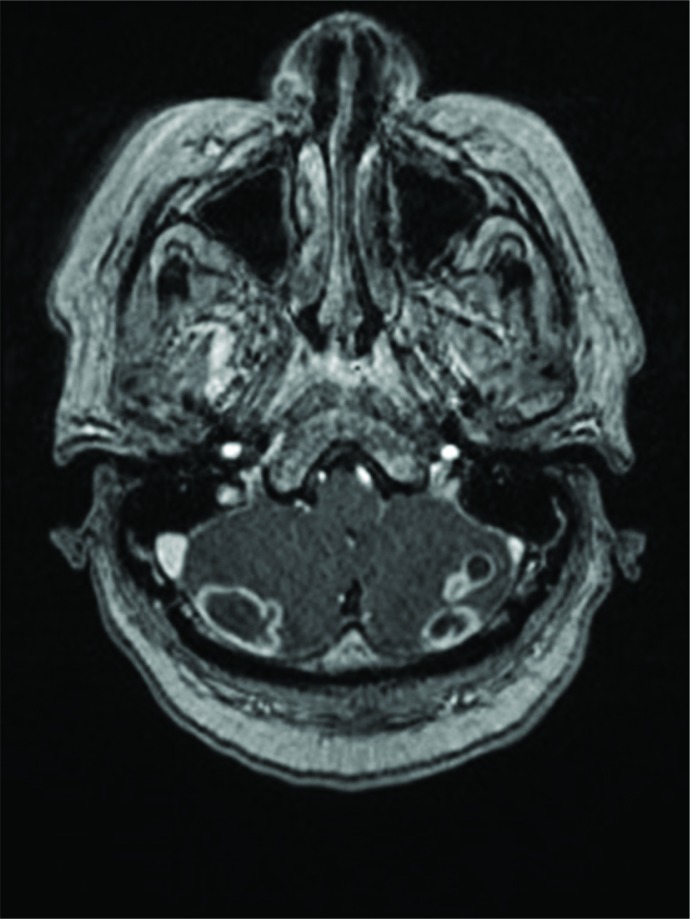
MRI of patient showing multiple ring-enhanced lesions in the cerebellum.

Initial laboratory results included a normal white blood cell (WBC) count of 7.9. The patient underwent a head computed tomography (CT), which showed a heterogenous density of the cerebellum and a subsequent magnetic resonance imaging (MRI) of the brain, which confirmed multiple ring-enhancing lesions of the inferior cerebellum with four discrete lesions (Fig. 1).

The patient was empirically started on treatment for presumptive toxoplasmosis with pyrimethamine, sulfadiazene, and folinic acid. In confirmatory testing, however, 2 days later, Toxoplasma IgM and IgG were reported negative and HIV was non-reactive. Therapy was discontinued.

On day 3, serum Cryptococcal antigen of 1:64 was reported. Treatment for cryptococcosis was initiated with liposomal amphotericin B 350 mg intravenously daily and flucytosine 2,500 mg orally every 6 hours. Head CTs were done every 2–3 days to assess brain edema and mass effect to determine if it would be safe to undertake a lumbar puncture. Dexamethasone was given, and after 24 hours, the patient reported complete resolution of headaches.

On day 11 of admission, repeat CT of the head showed decreased edema. Lumbar puncture was performed. Cerebrospinal fluid (CSF) analysis confirmed cryptococcal Ag with a titer of 1:4, WBC 230/mm^3^, lymphocytes 92%, and a negative culture. The patient completed 1 week of therapy whilst an inpatient with complete resolution of the presenting symptoms and was subsequently discharged with a 4-week course of amphotericin B and flucytosine. After completion of this regimen, the patient was started on maintenance therapy with fluconazole and continued while tapering off the steroids.

## Etiology

Cryptococcal infection is the fourth most common opportunistic infection seen in AIDS patients with CD4 counts <100. The use of chronic steroids accounts for about 1/3 non-HIV-related cryptococcal infections (2). *C. neoformans*, a saprophyte, is commonly found in soil contaminated with pigeon droppings, but has also been isolated from the wood of several tree species in South America and India, including the flowers and bark of eucalyptus trees (3, 4). Inhalation of small encapsulated yeasts may lead to an initial pulmonary infection; however, this is usually short lived and frequently silent, especially in immunocompetent individuals (3). The infection may follow three routes after the initial pulmonary infection, again depending usually on the person's immune status; contained within granulomata as a latent infection, disseminates, or is effectively cleared. The few in whom disease disseminates typically have defects in T-cell function (2–4), which include those immunosuppressed by either HIV, chronic steroids, or other immune suppressing agents, malignancy, or other chronic inflammatory diseases. The common finding that cryptococcomas are rarely seen in those with HIV supports the role of deficient cell-mediated immunity, notably altered macrophage and microglial function (2).

*C. neoformans* is the most common fungus to cause meningitis. The CSF is an ideal site for infection as it lacks complements and immunoglobulins. *C. neoformans* is an intracellular as well as an extracellular pathogen. This fungi's propensity for survival helps it remain for at least 18 months in interstitial granulomata (2). The ability for *C. neoformans* to survive relates to the potency and effectiveness of its virulence factors; the capacity to grow at 37°C, its capsule (which is antiphagocytic and down-regulates cellular and humoral immune responses when shed into host tissues), and lactase and melanin that interfere with oxidative killing by phagocytes.

## Diagnosis

Suspicion of cryptococcal neurologic infection can be difficult due to the non-specific presentation and symptoms of infected patients. Lumbar puncture is essential to establish the diagnosis. This might be delayed if the patient is experiencing focal neurological deficits, altered mental status, or papilledema. In the case of focal neurological signs, a lumbar puncture should be obtained after the appropriate radiological studies. CT and MRI are commonly used radiographic studies in patients with suspected cryptococcal meningitis. MRI is more sensitive in detecting cryptococcal CNS infections such as leptomeningeal enhancement and Virchow-Robin space dilatation (5). The gold standard of diagnosis is a positive growth of *C. neoformans* in the CSF, which is positive in almost all cases of cryptococcal infections. Serum cryptococcal antigen in non-HIV patients should not be used due to its low sensitivity.

## Treatment

The cornerstone of effective clearing of cryptococcal infection involves three phases to treatment: induction, consolidation, and maintenance. The time course for treatment at each of these stages differ depending on the etiology of the host's immunocompromised state, either as HIV-infected, transplant recipients, and non-HIV, non-transplant patients (6). The induction stage is usually longer for the non-HIV, non-transplant patients and the maintenance therapy for HIV-infected patients is longer, as it should be continued for at least a year and then longer if the CD4 count is still not >100. Each of the three groups includes the same antifungals; however, the precluding cause to their immunosuppressive state must also be addressed during this treatment course.

Amphotericin B (AmBd), flucytosine, and fluconazole remain the ideal first-choice combination for antifungals and are recommendations based on class A-I to B-II evidence per IDSA's systematic review (6). Induction therapy involves the most potent and most toxic combination so drug and side effect profile monitoring are essential during this phase. AmBd and flucytosine are administered for 2–4 weeks but the course of treatment is ultimately dependent on negative lumbar punctures and resolving symptoms.

Follow-up is essential for the complete eradication of the cryptococcal infection. Monitoring with serial MRIs is important to ensure resolution of cryptococcomas, if present, as they may represent other infectious or neoplastic causes of ring-enhancing lesions. Lumbar punctures every 2 weeks during the induction phase should be performed till the CSF becomes sterile. This will dictate the length of the induction phase as the timing is reset every time the CSF has cryptococcal growth.
